# Causal association of blood metabolites, immune cells, and lung cancer: A mediation Mendelian randomization study

**DOI:** 10.1097/MD.0000000000042053

**Published:** 2025-04-04

**Authors:** Tonglin Sun, Sheng Chen, Zhengyi Liu, Yuxiang Hu, Yinhui Sun, Lihuai Wang

**Affiliations:** a Department of Oncology, The First Hospital of Hunan University of Chinese Medicine, Changsha, Hunan Province, P.R. China; b Medical College, Hunan University of Chinese Medicine (Pathophysiology), Changsha, Hunan Province, P.R. China.

**Keywords:** blood metabolites, immune cells, Mendelian randomization, non-small cell lung cancer, small cell lung cancer

## Abstract

Lung cancer ranks highest in annual mortality among all cancers, and blood metabolites may influence its onset and progression. Our objective is to assess the causal relationships between blood metabolites and both non-small cell lung cancer (NSCLC) and small cell lung cancer (SCLC), while exploring the mediating effects of immune cells. We utilized publicly available genetic data to investigate the potential causal relationships between blood metabolites and NSCLC as well as SCLC using a 2-sample Mendelian randomization (MR) approach. In our primary analysis, we employed the inverse variance weighted (IVW) method and conducted sensitivity analyses and Steiger test to assess the reliability and directionality of the causal relationships. Additionally, we employed a 2-step MR approach to evaluate the mediating role of immune cells in these causal relationships. The IVW method revealed that palmitoylcarnitine levels (metabolon platform) and 4 other blood metabolites are risk factors for NSCLC, while tetrahydrocortisol glucuronide levels and 2 other blood metabolites are protective factors for NSCLC. Additionally, Alpha-hydroxyisovalerate levels and 8 other blood metabolites are risk factors for SCLC, whereas dimethylglycine levels and 3 other blood metabolites are protective factors for SCLC. Furthermore, IgD^-^ CD27^-^ B cell %B cell, CD27 on IgD^-^ CD38dim B cell, and CD3 on Naive CD4^+^ T cell mediate some of the relationships between blood metabolites and NSCLC. Activated and secreting CD4 regulatory T cell %CD4^+^ T cell, CD14^-^ CD16^-^ Absolute Count, and IgD on IgD^+^ CD24^+^ B cell mediate some of the relationships between blood metabolites and SCLC. There are significant causal relationships between blood metabolites and both NSCLC and SCLC, with some of these relationships mediated by immune cells. This aids us in influencing the role of blood metabolites in lung cancer by intervening with immune cells, thereby providing more avenues for the prevention and treatment of lung cancer.

## 1. Introduction

In most countries, despite a declining trend in the mortality rate of lung cancer, it remains a leading cause of cancer-related deaths.^[1]^ Classified histologically into non-small cell lung cancer (NSCLC) and small cell lung cancer (SCLC), they represent approximately 76% and 13% of lung cancer cases in the United States, respectively.^[2]^ From 1990 to 2021, the global burden of lung cancer has shown an overall downward trend.^[3]^ With the advent of immunotherapy and targeted therapy, there has been significant improvement in the treatment of NSCLC, resulting in a continuous decline in overall mortality rates among patients. However, the efficacy of treatment for SCLC remains limited. Additionally, due to the lack of early screening techniques, many patients are diagnosed with advanced or locally metastatic lung cancer upon initial detection. Therefore, it is imperative to identify effective approaches for screening and treating both NSCLC and SCLC.

Blood metabolites are small molecular products of natural metabolic reactions within the human body, widely present in the bloodstream and influenced by various factors such as lifestyle, dietary habits, genetic predisposition, and diseases. They serve as indicators of physiological and pathological states and can influence the onset and progression of diseases. Research has shown a significant association between blood metabolites and the gut microbiota.^[4]^ Many blood metabolites have become clinical markers for predicting diseases, and research has utilized them to construct predictive models for multiple diseases, providing valuable clinical guidance.^[5]^ Blood metabolites are also closely related to the construction of the tumor microenvironment, cancer cell proliferation, and signal transduction.^[6]^ Furthermore, research has established metabolite analysis models based on plasma proteomics to evaluate the efficacy and recurrence of NSCLC.^[7]^ Therefore, blood metabolites hold the potential to become targets for disease treatment interventions. Exploring the causal relationship between blood metabolites and lung cancer can provide valuable assistance in targeted lung cancer therapy.

The immune system is crucial for human health, with immune cells serving as the primary executors of immune function, capable of identifying and clearing cancer cells through various pathways. However, as tumors develop, the body’s immune system becomes suppressed, leading to immune escape by cancer cells, immune tolerance, and promotion of cancer cell proliferation, exacerbating the condition. Immunotherapy for cancer has emerged as a hotspot in medical development, aiming to restore normal antitumor immune responses in the body and control and eliminate cancer cells.^[8]^ The efficacy of CAR-T cell therapy and CAR-NK cell therapy in the field of cancer treatment has been validated.^[9,10]^ A recent pan-cancer analysis has identified novel biomarkers, such as EPHB2, which could play a key role in predicting prognosis and guiding immunotherapy responses for lung cancer patients.^[11]^ Additionally, a comprehensive study on the solute carrier family 35 member A2 emphasizes its clinical implications and potential value for immunotherapy in various cancers, including lung cancer.^[12]^ These findings suggest that personalized treatment strategies targeting specific biomarkers could improve therapeutic outcomes for lung cancer patients. Therefore, finding safe and effective immunotherapy approaches holds immense exploration potential. Research indicates that gut microbiota metabolites can enhance cancer treatment outcomes by modulating immune cell immunity.^[13,14]^ Similarly, there should be a similar relationship between blood metabolites and immune cells, thus impacting cancer development. Therefore, exploring the mediating role of immune cells in the relationship between blood metabolites and lung cancer is worth investigating.

Mendelian randomization (MR) is a method that uses genetic variation as instrumental variables (IVs) to infer causal relationships between exposure factors and outcome factors. By combining MR with mediation analysis, it enhances causal inference in mediation analysis, preventing bias in the relationships between exposure factors, mediator variables, and outcome factors due to confounding factors.^[15]^ Currently, research on the impact of blood metabolites through immune cells on the progression of NSCLC and SCLC is incomplete, and their causal relationships have not been fully explored. Therefore, this study utilizes MR to assess the potential causal relationships between blood metabolites and NSCLC as well as SCLC and investigates the mediating effects of immune cells therein.

## 2. Materials and methods

### 2.1. Study design

**Figure 1.**
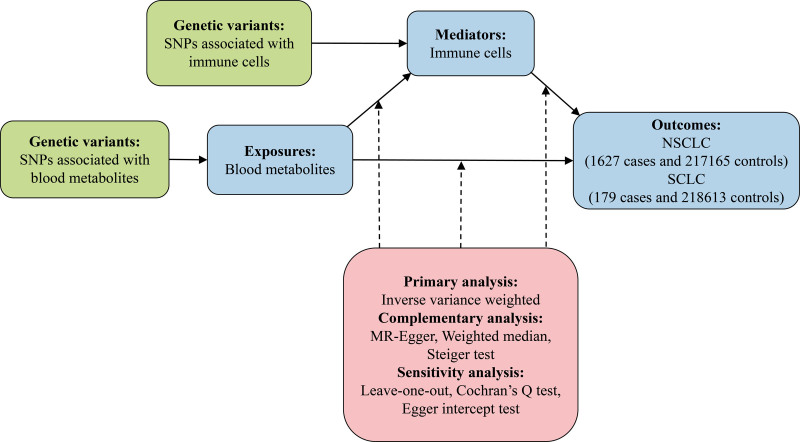
The design of 2-step MR study of blood metabolites on NSCLC and SCLC mediated by immune cells. MR = Mendelian randomization, NSCLC = non-small cell lung cancer, SCLC = small cell lung cancer, SNPs = single-nucleotide polymorphisms.

In this study, we employed a 2-step MR approach, utilizing various statistical methods and sensitivity analyses, to assess the causal relationships between blood metabolites and both NSCLC and SCLC, as well as to determine whether immune cells act as mediators. Initially, 2-sample MR analysis was conducted to investigate the relationships between blood metabolites and both NSCLC and SCLC, resulting in the identification of blood metabolites strongly correlated with lung cancer. Similarly, 2-sample MR analysis was performed to examine the relationships between immune cells and both NSCLC and SCLC, resulting in the selection of immune cells strongly associated with lung cancer. Subsequently, the causal effects of the selected blood metabolites on the selected immune cells were analyzed. Finally, specific immune cells’ mediating effects and mediation proportions in the causal relationships between specific blood metabolites and both NSCLC and SCLC were determined. Figure 1 illustrates the entire study design.

### 2.2. Data sources

The exposure factors are derived from a recent genetic study based on Canadian population statistics concerning plasma metabolomic profiles,^[16]^ utilizing Genome-Wide Association Study (GWAS) IDs (GCST90199621–GCST90201020). This study encompasses 1091 blood metabolites and 309 metabolite ratios. The mediator factors are obtained from a recent genetic study on immune cell characteristics in European populations,^[17]^ also utilizing GWAS IDs provided by the authors (GCST0001391–GCST0002121), which include 731 immune cell traits. Data for NSCLC (including 1627 cases and 217,165 controls) and SCLC (including 179 cases and 218,613 controls) were sourced from the IEU GWAS database (https://gwas.mrcieu.ac.uk/), with GWAS IDs Finn-b-C3_LUNG_NONSMALL and Finn-b-C3_SCLC respectively. These data are all publicly available, thus no additional ethical approval is required for this study.

### 2.3. Selection of IVs

For genetic variation to serve as effective IVs, 3 key assumptions must be met: strong correlation between genetic variation and blood metabolites or immune cells, absence of correlation between genetic variation and other potential confounding factors, and genetic variation does not affect NSCLC or SCLC through variables other than blood metabolites and immune cells.^[18]^

To ensure these criteria were met, we initially selected single-nucleotide polymorphisms (SNPs) that achieved genome-wide significance (GWS) with a *P*-value < 5 × 10^−8^, thereby ensuring a strong correlation between the SNPs and the exposure factors (blood metabolites or immune cells). To account for linkage disequilibrium, we applied a clumping procedure with a threshold of *r*^2^ < 0.001 within a 10,000-kb window, thus minimizing bias from correlated SNPs.

Next, SNPs directly associated with outcome factors (*P*-value < 5 × 10^−8^) were excluded to avoid pleiotropy-related confounding. The strength of the remaining SNPs as IVs was assessed using F-statistics, calculated as $F=\frac{R^{2}\left(n-k-1\right)}{\left(1-R^{2}\right)k}$, where *R*^2^ represents the proportion of variance in the exposure explained by the IVs, *n* is the sample size, and *k* is the number of instruments. SNPs with *F*-statistics > 10 were retained to eliminate weak instruments, as weak instruments can lead to biased causal estimates. Comprehensive information on selected IVs is provided in Table S1, Supplemental Digital Content, https://links.lww.com/MD/O623.

### 2.4. Statistical analysis

We applied a suite of statistical methods to evaluate the causal relationships between exposures (blood metabolites or immune cells) and outcomes (NSCLC and SCLC). These included the inverse variance weighted (IVW) method, MR-Egger regression, and the weighted median (WM) method.

The IVW method was employed as the primary analytic approach, as it assumes no horizontal pleiotropy and leverages the reciprocal of outcome variances as weights for regression. This method provides efficient estimates when all IVs are valid. For robustness checks, MR-Egger regression, which allows for nonzero intercepts, was used to assess the presence of horizontal pleiotropy. A significant intercept term (*P*-value < .05) in MR-Egger regression would indicate potential horizontal pleiotropy.^[19]^ The WM method was also applied, as it yields consistent estimates even if up to 50% of the IVs are invalid, though at the cost of reduced precision.^[20]^

The directionality of causal relationships was confirmed using the Steiger test. This test verifies whether the genetic instrument affects the exposure before influencing the outcome. A *P*-value < .05 in the Steiger test suggests a unidirectional causal relationship with minimal risk of reverse causality.

To evaluate the reliability of results, sensitivity analyses were conducted, including Cochran *Q* test to detect heterogeneity among SNP effects (*P*-value < .05 indicates significant heterogeneity), MR-Egger intercept test to identify horizontal pleiotropy (*P*-value < .05 suggests pleiotropy), and leave-one-out analysis to assess the influence of individual SNPs on the overall causal estimate. Outlier SNPs disproportionately affecting the results were excluded from further analysis.

To investigate potential mediation effects, we conducted 2-step MR. This approach evaluates whether mediator factors (immune cells) explain part of the causal relationship between exposures (blood metabolites) and outcomes (lung cancer). The analysis began by performing 2-sample MR to estimate the total causal effect (*α*) of exposure factors on outcome factors. Subsequently, the causal effect (*β*1) of exposure factors on mediator factors was calculated using 2-sample MR, followed by another MR analysis to estimate the effect (*β*2) of mediator factors on outcome factors. The indirect effect (*β*3) was then determined as the product of *β*1 and *β*2. The mediation proportion was derived by dividing *β*3 by *α*, representing the fraction of the total effect explained by the mediator. During this process, SNPs associated with exposure factors were excluded when estimating mediator-outcome relationships to avoid confounding by direct associations.

All MR analyses mentioned above were conducted using R version 4.3.1 (https://www.r-project.org) and the “2-sample MR” R package.

## 3. Result

### 3.1. The total effect of blood metabolites on NSCLC and SCLC individually

**Figure 2.**
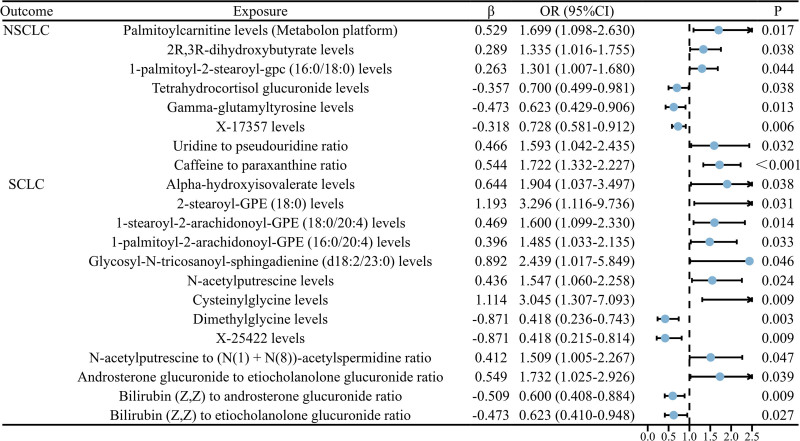
The forest plot displays the causal relationship between blood metabolites and both NSCLC and SCLC. CI = confidence interval, NSCLC = non-small cell lung cancer, OR = odds ratio, SCLC = small cell lung cancer.

Through 2-sample MR analysis of the causal relationship between blood metabolites and NSCLC, palmitoylcarnitine levels (metabolon platform) (OR = 1.699, 95% CI = 1.098–2.630, *P* = .017), 2R,3R-dihydroxybutyrate levels (OR = 1.335, 95% CI = 1.016–1.755, *P* = .038), 1-palmitoyl-2-stearoyl-gpc (16:0/18:0) levels (OR = 1.301, 95% CI = 1.007–1.680, *P* = .044), Uridine to pseudouridine ratio (OR = 1.593, 95% CI = 1.042–2.435, *P* = .032), and Caffeine to paraxanthine ratio (OR = 1.722, 95% CI = 1.332–2.227, *P* < .001) were identified as risk factors for NSCLC. Tetrahydrocortisol glucuronide levels (OR = 0.700, 95% CI = 0.499–0.981, *P* = .038), Gamma-glutamyltyrosine levels (OR = 0.623, 95% CI = 0.429–0.906, *P* = .013), and X-17357 levels (OR = 0.728, 95% CI = 0.581–0.912, *P* = .006) were identified as protective factors for NSCLC. The results are illustrated in Figure 2. Additionally, the Steiger test revealed that palmitoylcarnitine levels (metabolon platform), Tetrahydrocortisol glucuronide levels, Gamma-glutamyltyrosine levels, and Uridine to pseudouridine ratio exhibit bidirectional causal relationships with NSCLC.

Through 2-sample MR analysis of the causal relationship between blood metabolites and SCLC, the following were identified as risk factors for SCLC: alpha-hydroxyisovalerate levels (OR = 1.904, 95% CI = 1.037–3.497, *P* = .038), 2-stearoyl-GPE (18:0) levels (OR = 3.296, 95% CI = 1.116–9.736, *P* = .031), 1-stearoyl-2-arachidonoyl-GPE (18:0/20:4) levels (OR = 1.600, 95% CI = 1.099–2.330, *P* = .014), 1-palmitoyl-2-arachidonoyl-GPE (16:0/20:4) levels (OR = 1.485, 95% CI = 1.033–2.135, *P* = .033), glycosyl-N-tricosanoyl-sphingadienine (d18:2/23:0) levels (OR = 2.439, 95% CI = 1.017–5.849, *P* = .046), N-acetylputrescine levels (OR = 1.547, 95% CI = 1.060–2.258, *P* = .024), Cysteinylglycine levels (OR = 3.045, 95% CI = 1.307–7.093, *P* = .009), N-acetylputrescine to (N(1) + N(8))-acetylspermidine ratio (OR = 1.509, 95% CI = 1.005–2.267, *P* = .047), and Androsterone glucuronide to etiocholanolone glucuronide ratio (OR = 1.732, 95% CI = 1.025–2.926, *P* = .039). Additionally, the following were identified as protective factors for SCLC: dimethylglycine levels (OR = 0.418, 95% CI = 0.236–0.743, *P* = .003), X-25422 levels (OR = 0.418, 95% CI = 0.215–0.814, *P* = .009), bilirubin (Z,Z) to androsterone glucuronide ratio (OR = 0.600, 95% CI = 0.408–0.884, *P* = .009), and bilirubin (Z,Z) to etiocholanolone glucuronide ratio (OR = 0.623, 95% CI = 0.410–0.948, *P* = .027). The results are depicted in Figure 2. Additionally, the Steiger test revealed bidirectional causal relationships between 2-stearoyl-GPE (18:0) levels and glycosyl-N-tricosanoyl-sphingadienine (d18:2/23:0) levels with SCLC.

### 3.2. The impact of immune cells on NSCLC and SCLC

**Figure 3.**
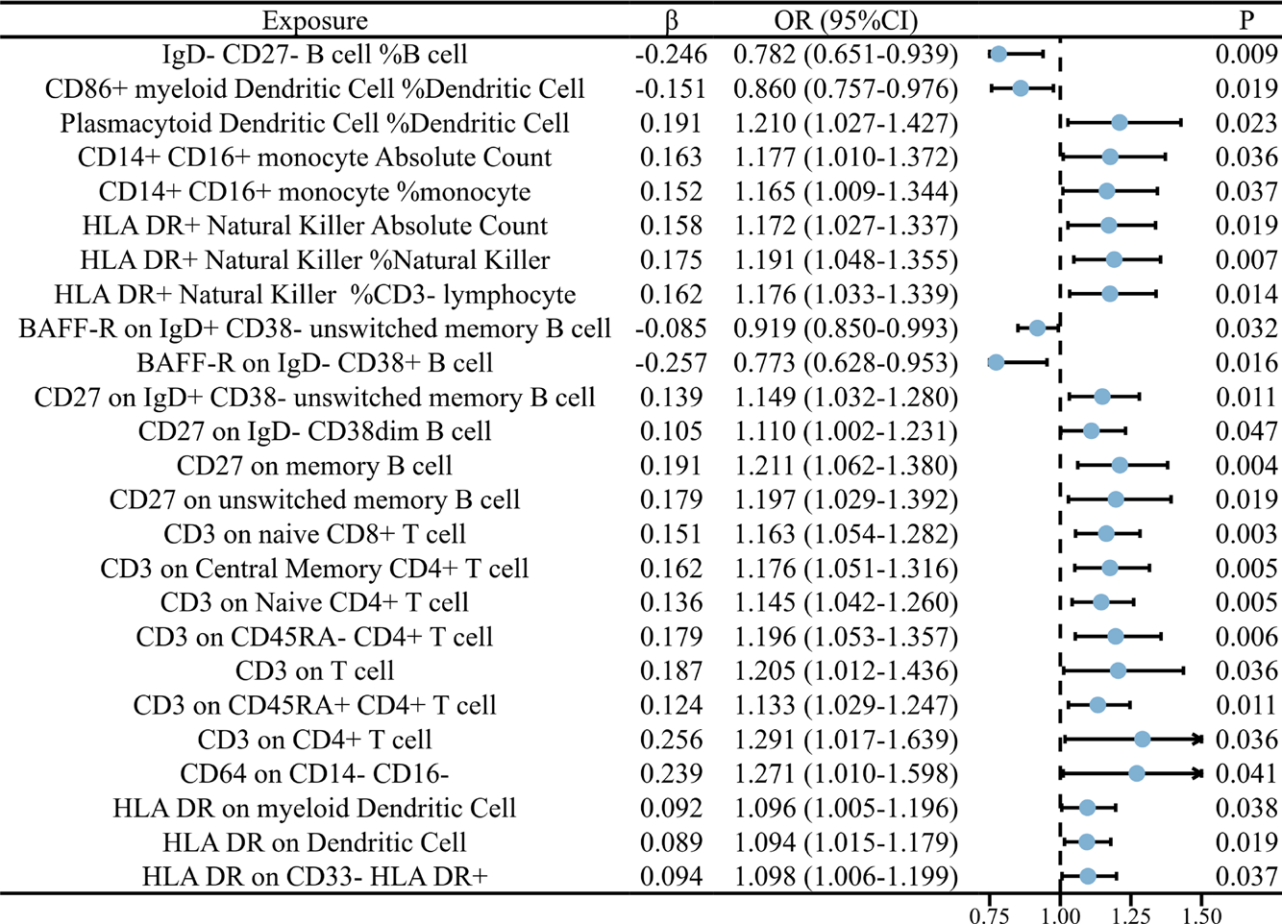
The forest plot displays the causal relationship between immune cells and NSCLC. CI = confidence interval, NSCLC = non-small cell lung cancer, OR = odds ratio.

Using the IVW method, the following immune cell factors were identified as risk factors for NSCLC: plasmacytoid dendritic cell %dendritic cell (OR = 1.210, 95% CI = 1.027–1.427, *P* = .023), CD14^+^ CD16^+^ monocyte absolute count (OR = 1.177, 95% CI = 1.010–1.372, *P* = .036), CD14^+^ CD16^+^ monocyte %monocyte (OR = 1.165, 95% CI = 1.009–1.344, *P* = .037), HLA DR^+^ natural killer absolute count (OR = 1.172, 95% CI = 1.027–1.337, *P* = .019), HLA DR^+^ natural killer %natural killer (OR = 1.191, 95% CI = 1.048–1.355, *P* = .007), HLA DR^+^ natural killer %CD3^-^ lymphocyte (OR = 1.176, 95% CI = 1.033–1.339, *P* = .014), CD27 on IgD^+^ CD38^-^ unswitched memory B cell (OR = 1.149, 95% CI = 1.032–1.280, *P* = .011), CD27 on IgD^-^ CD38dim B cell (OR = 1.110, 95% CI = 1.002–1.231, *P* = .047), CD27 on memory B cell (OR = 1.211, 95% CI = 1.062–1.380, *P* = .004), CD27 on unswitched memory B cell (OR = 1.197, 95% CI = 1.029–1.392, *P* = .019), CD3 on naive CD8^+^ T cell (OR = 1.163, 95% CI = 1.054–1.282, *P* = .003), CD3 on central memory CD4^+^ T cell (OR = 1.176, 95% CI = 1.051–1.316, *P* = .005), CD3 on Naive CD4^+^ T cell (OR = 1.145, 95% CI = 1.042–1.260, *P* = .005), CD3 on CD45RA^-^ CD4^+^ T cell (OR = 1.196, 95% CI = 1.053–1.357, *P* = .006), CD3 on T cell (OR = 1.205, 95% CI = 1.012–1.436, *P* = .036), CD3 on CD45RA^+^ CD4^+^ T cell (OR = 1.133, 95% CI = 1.029–1.247, *P* = .011), CD3 on CD4^+^ T cell (OR = 1.291, 95% CI = 1.017–1.639, *P* = .036), CD64 on CD14^-^ CD16^-^ (OR = 1.271, 95% CI = 1.010–1.598, *P* = .041), HLA DR on myeloid dendritic cell (OR = 1.096, 95% CI = 1.005–1.196, *P* = .038), HLA DR on dendritic cell (OR = 1.094, 95% CI = 1.015–1.179, *P* = .019), HLA DR on CD33^-^ HLA DR^+^ (OR = 1.098, 95% CI = 1.006–1.199, *P* = .037). Conversely, IgD^-^ CD27- B cell %B cell (OR = 0.782, 95% CI = 0.651–0.939, *P* = .009), CD86^+^ myeloid dendritic cell %dendritic cell (OR = 0.860, 95% CI = 0.757–0.976, *P* = .019), BAFF-R on IgD^+^ CD38^-^ unswitched memory B cell (OR = 0.919, 95% CI = 0.850–0.993, *P* = .032), and BAFF-R on IgD^-^ CD38^+^ B cell (OR = 0.773, 95% CI = 0.628–0.953, *P* = .016) were identified as protective factors for NSCLC. The results are illustrated in Figure 3. Furthermore, the Steiger test revealed bidirectional causal relationships between CD64 on CD14^-^ CD16^-^ and NSCLC.

**Figure 4.**
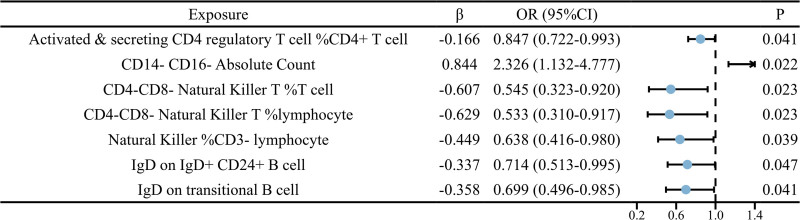
The forest plot displays the causal relationship between immune cells and SCLC. CI = confidence interval, OR = odds ratio, SCLC = small cell lung cancer.

Using the IVW method, CD14^-^ CD16^-^ absolute count (OR = 2.326, 95% CI = 1.132–4.777, *P* = .022) was identified as a risk factor for SCLC. Conversely, activated and secreting CD4 regulatory T cell %CD4^+^ T cell (OR = 0.847, 95% CI = 0.722–0.993, *P* = .041), CD4^-^CD8^-^ Natural Killer T %T cell (OR = 0.545, 95% CI = 0.323–0.920, *P* = .023), CD4^-^CD8^-^ Natural Killer T %lymphocyte (OR = 0.533, 95% CI = 0.310–0.917, *P* = .023), natural killer %CD3^-^ lymphocyte (OR = 0.638, 95% CI = 0.416–0.980, *P* = .039), IgD on IgD^+^ CD24^+^ B cell (OR = 0.714, 95% CI = 0.513–0.995, *P* = .047), and IgD on transitional B cell (OR = 0.699, 95% CI = 0.496–0.985, *P* = .041) were identified as protective factors for SCLC. The results are depicted in Figure 4. Additionally, the Steiger test revealed bidirectional causal relationships between CD14^−^ CD16^−^ absolute count and natural killer %CD3^-^ lymphocyte with SCLC.

### 3.3. The impact of blood metabolites on immune cells

Using MR analysis with blood metabolites and immune cells causally related to NSCLC, it was found that Tetrahydrocortisol glucuronide levels are positively associated with IgD^−^ CD27^−^ %B cell (OR = 1.395, 95% CI = 1.089–1.787, *P* = .008), X-17357 levels are positively associated with CD27 on IgD^-^ CD38dim (OR = 1.177, 95% CI = 1.006–1.378, *P* = .042), and X-17357 levels are negatively associated with CD3 on Naive CD4^+^ (OR = 0.823, 95% CI = 0.692–0.979, *P* = .028). Moreover, all of these relationships are bidirectional causal associations.

**Figure 5.**
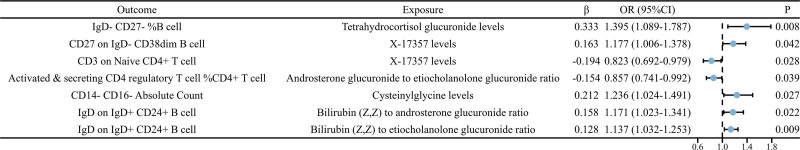
The forest plot displays the causal relationship between blood metabolites and immune cells. CI = confidence interval, OR = odds ratio.

Utilizing MR analysis with blood metabolites and immune cells causally related to SCLC, it was observed that the ratio of androsterone glucuronide to etiocholanolone glucuronide is negatively associated with activated and secreting Treg %CD4^+^ (OR = 0.857, 95% CI = 0.741–0.992, *P* = .039), cysteinylglycine levels are positively associated with CD14^−^ CD16^−^ AC (OR = 1.236, 95% CI = 1.024–1.491, *P* = .027), the ratio of bilirubin (Z,Z) to androsterone glucuronide is positively associated with IgD on IgD^+^ CD24^+^ (OR = 1.171, 95% CI = 1.023–1.341, *P* = .022), and the ratio of bilirubin (Z,Z) to etiocholanolone glucuronide is positively associated with IgD on IgD^+^ CD24^+^ (OR = 1.137, 95% CI = 1.032–1.253, *P* = .009). The results are illustrated in Figure 5. Additionally, Steiger test reveals bidirectional causal relationship between cysteinylglycine levels and CD14^-^ CD16^-^ AC.

### 3.4. The mediating role of immune cells in the relationship between blood metabolites and both NSCLC and SCLC

**Figure 6.**
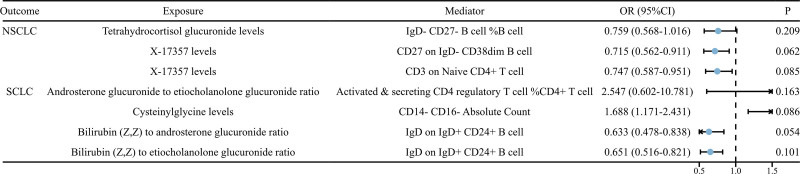
The forest plot displays the causal relationships between blood metabolites and NSCLC and SCLC after removing the mediating effects of immune cells. CI = confidence interval, NSCLC = non-small cell lung cancer, OR = odds ratio, SCLC = small cell lung cancer.

Through the above analysis combined with the 2-step MR analysis, we have determined that immune cells may mediate the causal relationships between 3 sets of blood metabolites and NSCLC, as well as between 4 sets of blood metabolites and SCLC. Figure 6 depicts the MR analysis results of these 7 causal relationships after removing the mediating effects. It can be observed that under the IVW model, all *P*-values are >.05, indicating that none of the causal relationships have statistical significance after removing the mediating effects. Therefore, it can be inferred that all 7 exposure factors influence the outcome factors through intermediate factors. The mediating proportion of IgD^-^ CD27^-^ B cell %B cell in the causal relationship between Tetrahydrocortisol glucuronide levels and NSCLC is 22.9%, the mediating proportion of CD27 on IgD^-^ CD38dim B cell in the causal relationship between X-17357 levels and NSCLC is −5.4%, the mediating proportion of CD3 on Naive CD4^+^ T cell in the causal relationship between X-17357 levels and NSCLC is 8.3%, the mediating proportion of Activated and secreting CD4 regulatory T cell %CD4^+^ T cell in the causal relationship between Androsterone glucuronide to etiocholanolone glucuronide ratio and SCLC is 4.7%, the mediating proportion of CD14^-^ CD16^-^ Absolute Count in the causal relationship between Cysteinylglycine levels and SCLC is 16.1%, the mediating proportion of IgD on IgD^+^ CD24^+^ B cell in the causal relationship between Bilirubin (Z,Z) to androsterone glucuronide ratio and SCLC is 10.5%, and the mediating proportion of IgD on IgD^+^ CD24^+^ B cell in the causal relationship between Bilirubin (Z,Z) to etiocholanolone glucuronide ratio and SCLC is 9.1%.

### 3.5. Sensitivity analyses

We also conducted sensitivity analysis on the causal relationships in this study (Table S2, Supplemental Digital Content, https://links.lww.com/MD/O624). Cochran *Q* test indicates that there is heterogeneity only in the causal relationship between 2R, 3R-dihydroxybutyrate levels and NSCLC, possibly due to biases caused by different sequencing methods or different sample populations. MR-Egger intercept test reveals no evidence of horizontal pleiotropy in any of the causal relationships. Leave-one-out analysis demonstrates that there is no single SNP dominating the overall causal relationships (Figs. S1–S5, Supplemental Digital Content, https://links.lww.com/MD/O625).

## 4. Discussion

Previous research has employed blood metabolites to analyze pathways in noncommunicable chronic diseases, suggesting the potential for early disease prevention through the observation of specific blood metabolites.^[21]^ Additionally, research indicates that plasma metabolomics can aid in the effective diagnosis and targeted prevention of metabolic syndrome.^[22]^ Blood metabolites are also closely linked to cancer, with studies analyzing their associations with cancer fatigue, early cancer screening, and monitoring.^[23–25]^ Cysteinylglycine, as a blood metabolite, is a pro-oxidant produced during the breakdown of glutathione metabolism, believed to induce lipid peroxidation and oxidative stress, leading to cancer progression. Some studies have shown that it is not significantly associated with breast cancer but negatively correlated with gastric cardia adenocarcinoma.^[26,27]^ Furthermore, research suggests that there are significant differences in plasma metabolite profiles between patients with multiple primary lung cancer and healthy individuals, indicating that blood metabolites may play a role in the occurrence and progression of lung cancer.^[28]^

To investigate the causal relationships between blood metabolites and both NSCLC and SCLC separately, we conducted 2-sample MR. We found that 5 sets of blood metabolite levels are risk factors for NSCLC, while 3 sets are protective factors. Additionally, 9 sets of blood metabolite levels are risk factors for SCLC, and 4 sets are protective factors. The immune system plays a crucial role in human disease resistance. To explore whether the aforementioned blood metabolites affect NSCLC and SCLC through immune cells, we employed 2-step MR. We found that 21 immune cell traits are risk factors for NSCLC, while 4 are protective factors. Furthermore, 1 immune cell trait is a risk factor for SCLC, and 6 are protective factors. Subsequently, we evaluated the causal relationships between the selected blood metabolites and immune cell traits. The results indicate that there are 7 sets of blood metabolites with causal relationships with immune cell traits. According to the study findings, IgD^-^ CD27^-^ B cell %B cell mediates the relationship between Tetrahydrocortisol glucuronide levels and the risk of NSCLC. CD27 on IgD^-^ CD38dim B cell and CD3 on Naive CD4^+^ T cell mediate the relationship between X-17357 levels and the risk of NSCLC. Activated and secreting CD4 regulatory T cell %CD4^+^ T cell mediates the relationship between Androsterone glucuronide to etiocholanolone glucuronide ratio and the risk of SCLC. CD14^−^ CD16^−^ Absolute Count mediates the relationship between Cysteinylglycine levels and the risk of SCLC. IgD on IgD^+^ CD24^+^ B cell mediates the relationship between Bilirubin (Z,Z) to androsterone glucuronide ratio and the risk of SCLC, while IgD on IgD^+^ CD24^+^ B cell mediates the relationship between Bilirubin (Z,Z) to etiocholanolone glucuronide ratio and the risk of SCLC.

The human immune system relies heavily on T cells and B cells. T cells play roles in both humoral and cellular immunity, while B cells primarily function in humoral immunity. Tumor immunotherapy often harnesses the functions of T cells and B cells to impact cancer. T cells are vital components of the immune system responsible for killing tumor cells. Research targeting T cells has revealed the existence of various T cell subsets with different functions, some of which promote while others inhibit the immune system. Their effective interaction protects the body from pathogens and tumors.^[29]^ Cancer immunotherapy targeting T cells has become a hot topic in cancer treatment, including immune checkpoint inhibitors (ICBs), adoptive cell therapy, and cancer vaccines, all of which are T cell-based immunotherapies.^[30,31]^ As 1 of the most important immune cells, B cells can differentiate into plasma cells to produce antibodies. Studies analyzing the composition of immune cells in the tumor microenvironment have confirmed a strong correlation between tumor-infiltrating B cells and antitumor efficacy and prognosis.^[32,33]^ Research in NSCLC has identified a large number of CD27^−^ IgD^−^ B cells, which negatively correlate with the presence of memory B cells within the tumor.^[34]^ B cells can also promote the differentiation of tumor-specific follicular helper CD4^+^ T cells, thereby playing a role in antitumor immune responses.^[35]^ Compared to traditional chemotherapy, immunotherapy has shown better efficacy and higher drug tolerance. Many new immunotherapy methods are continuously being explored through clinical trials.

Our findings align with previous research, such as the study by Xu et al, which employed a 2-sample bidirectional MR analysis to investigate the causal association between immune cells and lung cancer risk.^[36]^ Their study highlighted the significant role of immune cells in lung cancer development, supporting our observation that specific immune cell traits mediate the relationship between blood metabolites and lung cancer risk. However, our study extends these findings by incorporating a broader range of blood metabolites and employing a mediation analysis approach, providing a more comprehensive understanding of the interplay between blood metabolites, immune cells, and lung cancer.

The potential clinical implications of our findings are noteworthy. Identifying specific blood metabolites and immune cell traits that influence lung cancer risk opens avenues for developing targeted therapeutic strategies. For instance, interventions aimed at modulating the levels of harmful blood metabolites or altering the activity of specific immune cell subsets could be explored as potential treatments for lung cancer. Additionally, these biomarkers could serve as early detection tools, allowing for timely intervention and improved patient outcomes. Future research should focus on validating these findings in diverse populations and exploring the mechanistic pathways underlying these associations to facilitate the development of effective clinical applications.

This study has several strengths: Employing MR analysis, it reduces confounding biases based on simulated randomized controlled trials and employs various methods for sensitivity analysis to ensure the stability and reliability of the results. The 1091 blood metabolites, 309 metabolite ratios, 731 immune cell characteristics, as well as GWAS data for NSCLC and SCLC utilized in the study are derived from the latest and most comprehensive research and possess extensive genetic variation data. The screening of IVs is conducted with rigorous criteria, including setting GWS thresholds, *F*-statistic ranges, and linkage disequilibrium thresholds, to obtain genetic variations strongly associated with exposure and mediator factors, respectively.

This study has several limitations that should be acknowledged. Firstly, the genetic data were primarily derived from European populations, which may limit the generalizability of the findings to other ethnic groups. Differences in genetic architecture, environmental exposures, and lifestyle factors across populations could influence the observed causal relationships. Future studies should include data from more diverse populations, such as African and Asian cohorts, to validate these findings and enhance their global applicability. Secondly, the relatively small sample size for SCLC cases (n = 179) may reduce the statistical power, necessitating validation with larger datasets. Thirdly, despite rigorous sensitivity analyses, the possibility of horizontal pleiotropy influencing the results cannot be entirely excluded. Horizontal pleiotropy, where genetic variants affect the outcome through pathways other than the exposure of interest, could introduce biases into causal estimates. Future studies could utilize emerging methods, to better account for pleiotropy and assess its impact on causal inferences. Fourthly, potential bidirectional causal relationships among exposures, mediators, and outcomes may introduce biases in the mediation analysis, underscoring the importance of longitudinal data and refined statistical models. Lastly, while this study focused on immune cells as mediators, other unexplored mediators, such as metabolic pathways and environmental factors, warrant further investigation. Despite these limitations, this study provides valuable insights into the causal relationships between blood metabolites, immune cells, and lung cancer, offering a foundation for future research and therapeutic strategies.

## 5. Conclusion

This study combines MR analysis with mediation analysis to explore the potential causal relationships between blood metabolites and lung cancer (NSCLC and SCLC), identifying that some of these relationships are partially mediated by immune cells. These findings highlight the potential for interventions targeting immune cells to modulate the effects of blood metabolites on NSCLC and SCLC. However, the reliance on European datasets highlights the need for future research incorporating more diverse populations to ensure the broad applicability of these results. In addition, the possibility of other mediating factors in the causal pathway between blood metabolites and NSCLC or SCLC remains to be investigated. Comprehensive analyses that include a broader range of potential mediators and diverse populations will enhance our understanding of the complex mechanisms underlying lung cancer development and progression, offering new opportunities for targeted therapeutic strategies.

## Acknowledgments

The authors sincerely thank related investigators for sharing the GWAS summary statistics included in this study.

## Author contributions

**Data curation:** Tonglin Sun, Zhengyi Liu, Yuxiang Hu.

**Writing – original draft:** Tonglin Sun, Sheng Chen.

**Writing – review & editing:** Tonglin Sun, Yinhui Sun, Lihuai Wang.

## Supplementary Material


