# Supplementary material for: Causal association of blood metabolites, immune cells, and lung cancer: A mediation Mendelian randomization study
Source: Medicine (Baltimore). 2025 Apr 4;104(14):e42053. doi: 10.1097/MD.0000000000042053 (PMC11977704; doi:10.1097/MD.0000000000042053)

Figure S1. Leave-one-out analysis of the causal relationship between blood metabolites and NSCLC.


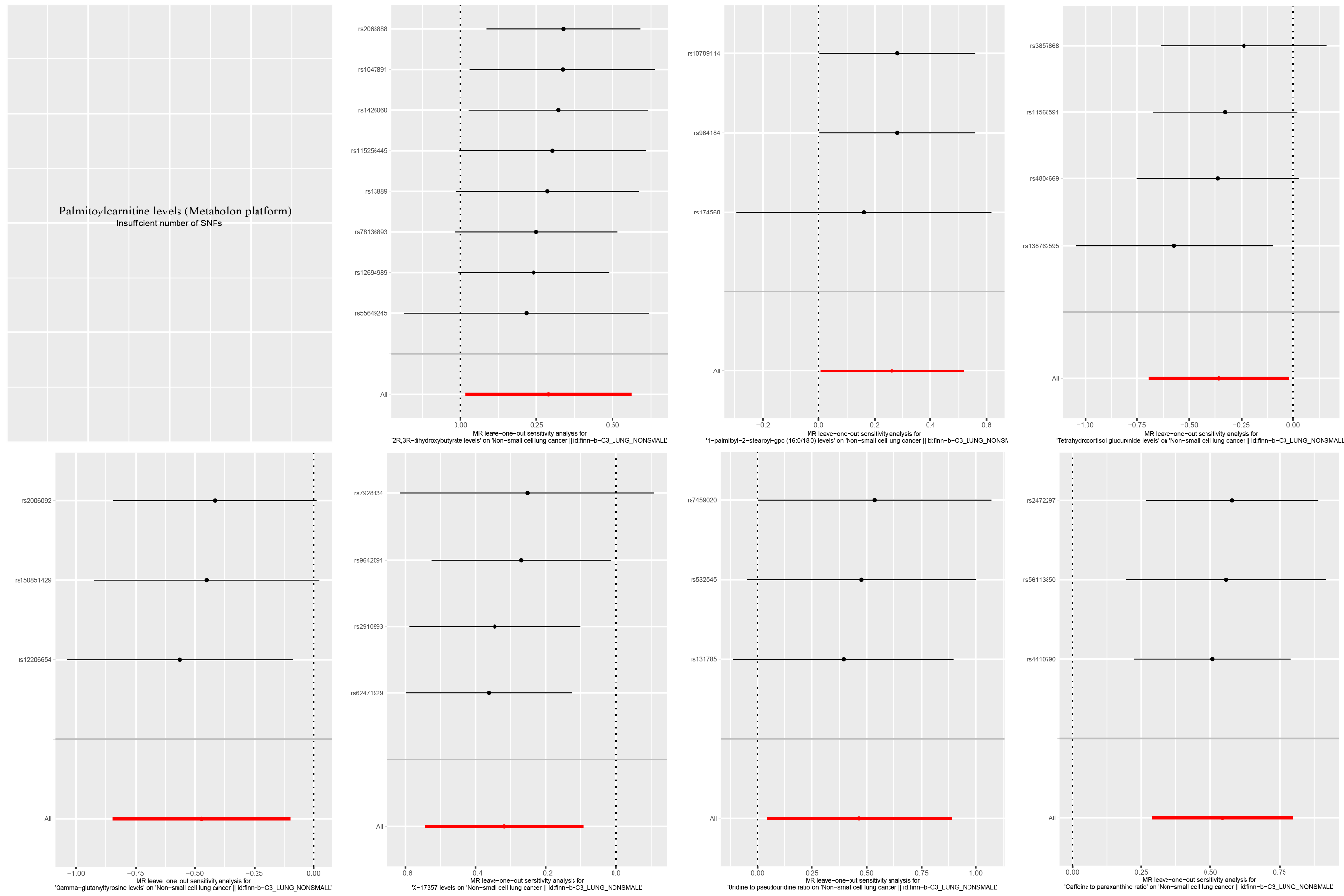


Figure S2. Leave-one-out analysis of the causal relationship between blood metabolites and SCLC.


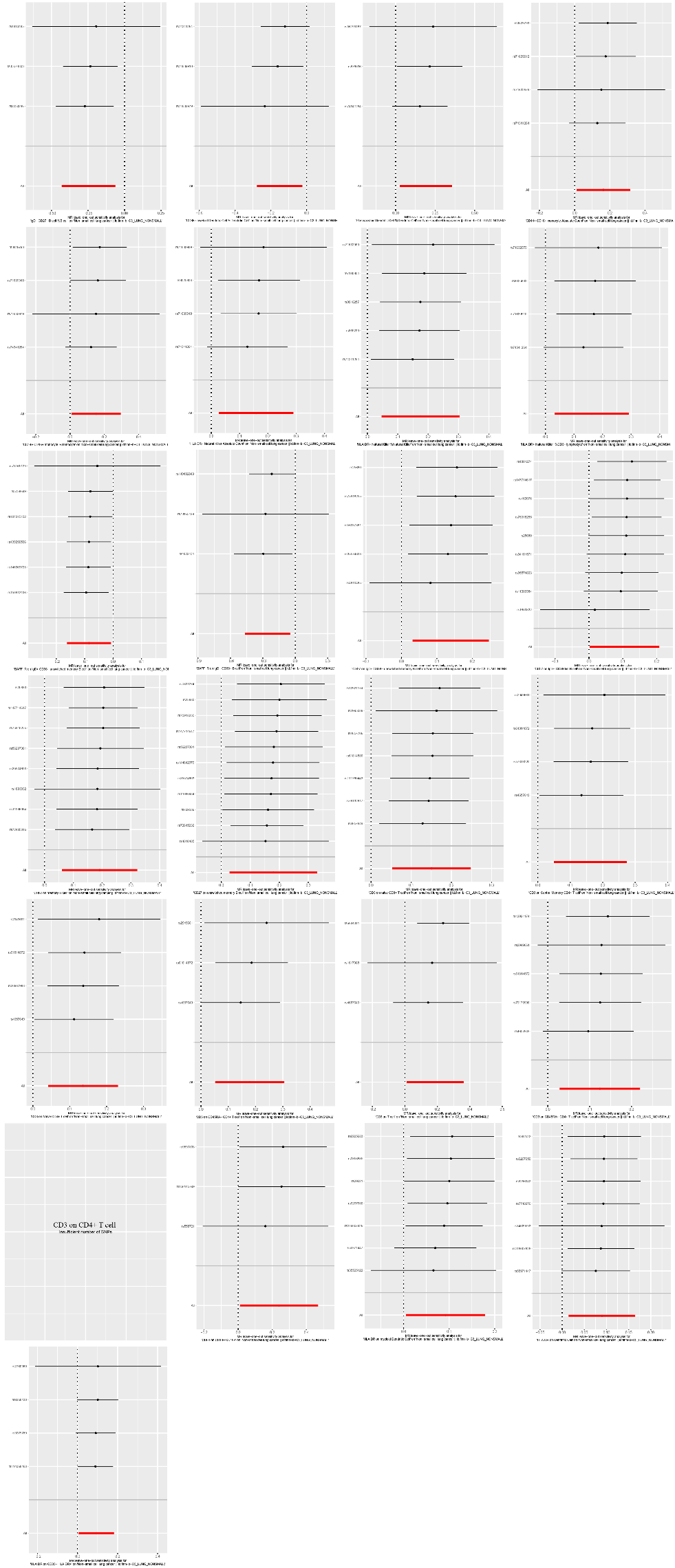


Figure S3. Leave-one-out analysis of the causal relationship between immune cells and NSCLC.


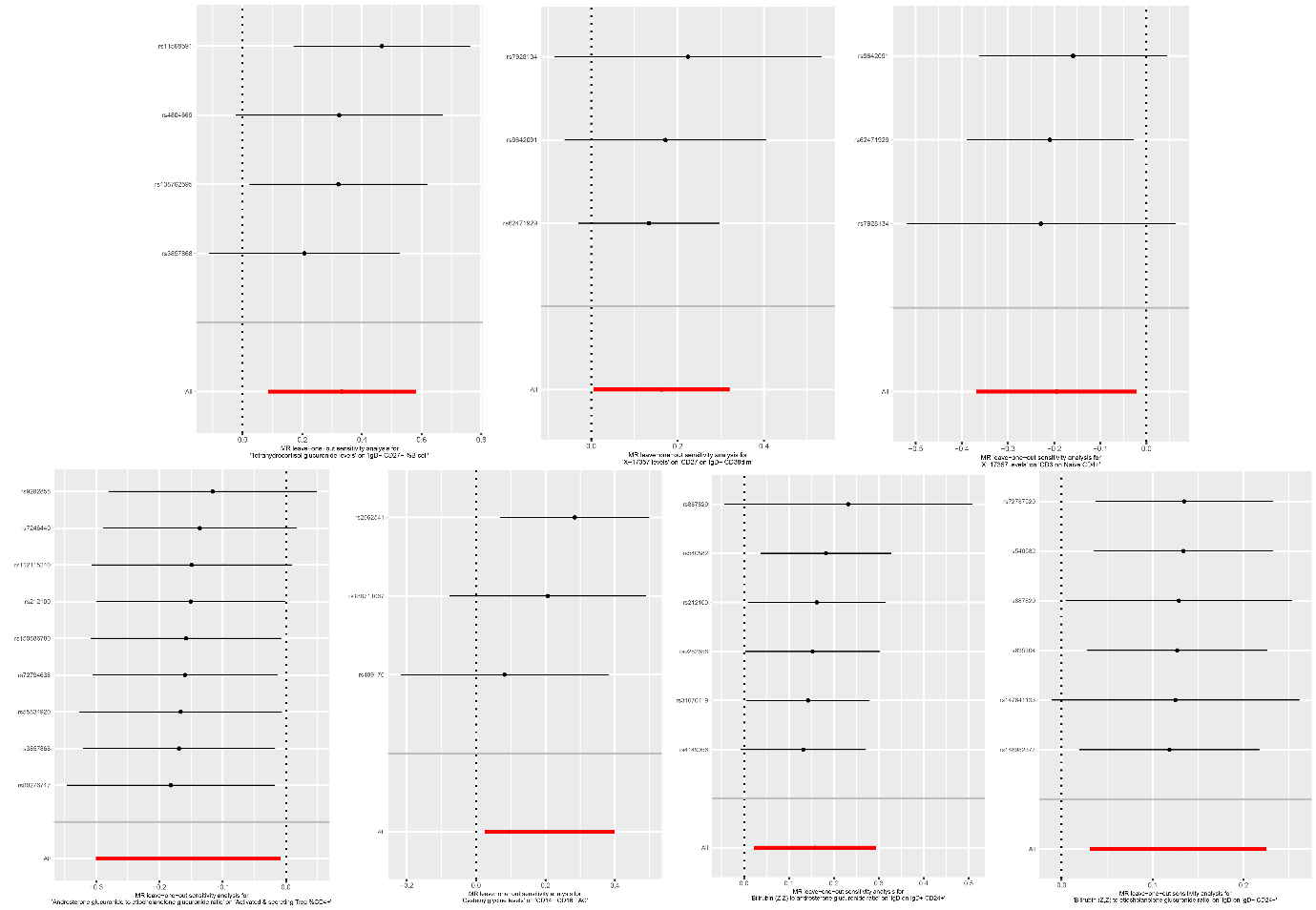


Figure S4. Leave-one-out analysis of the causal relationship between immune cells and SCLC.


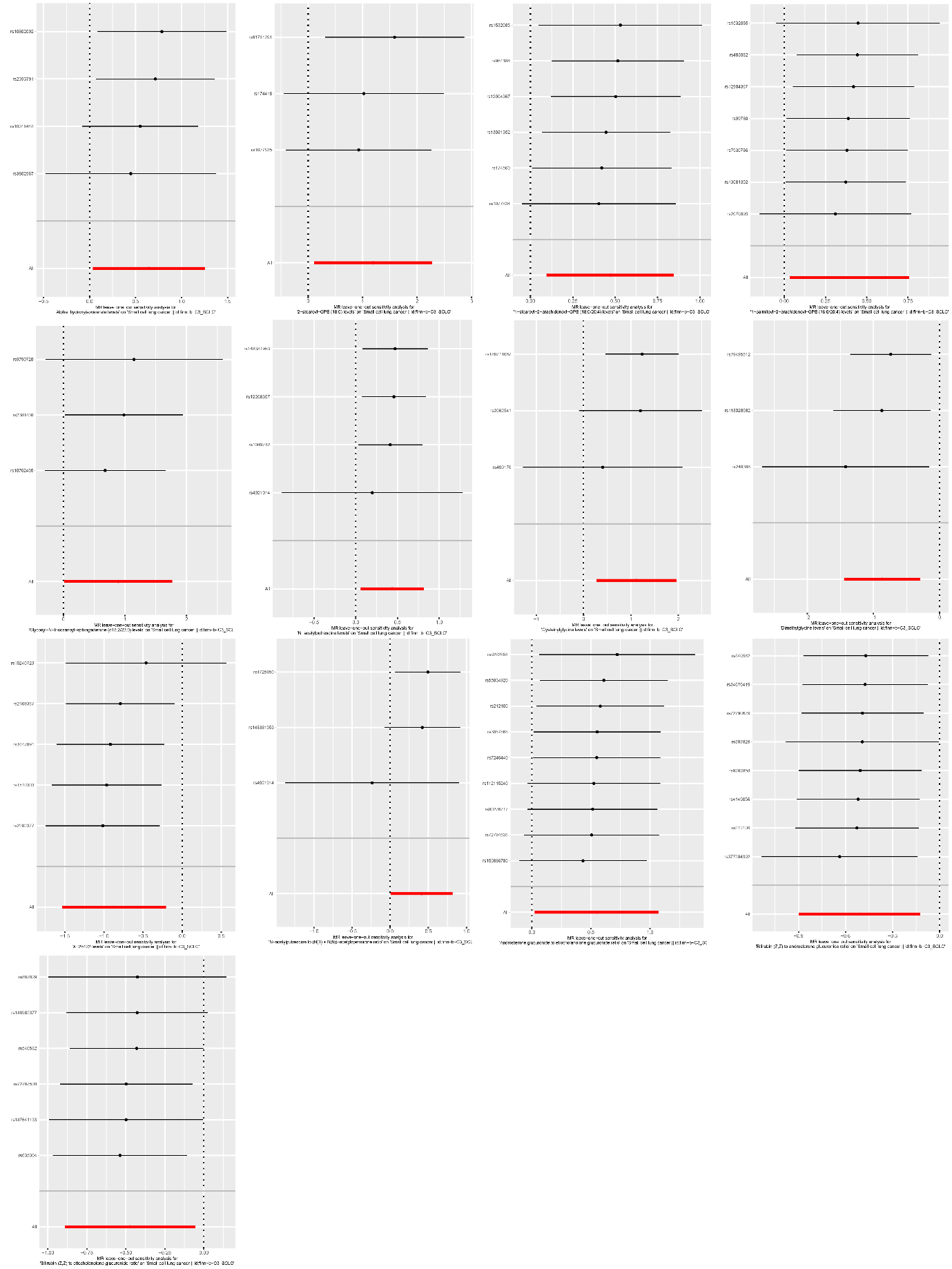


Figure S5. Leave-one-out analysis of the causal relationship between blood metabolites and immune cells.


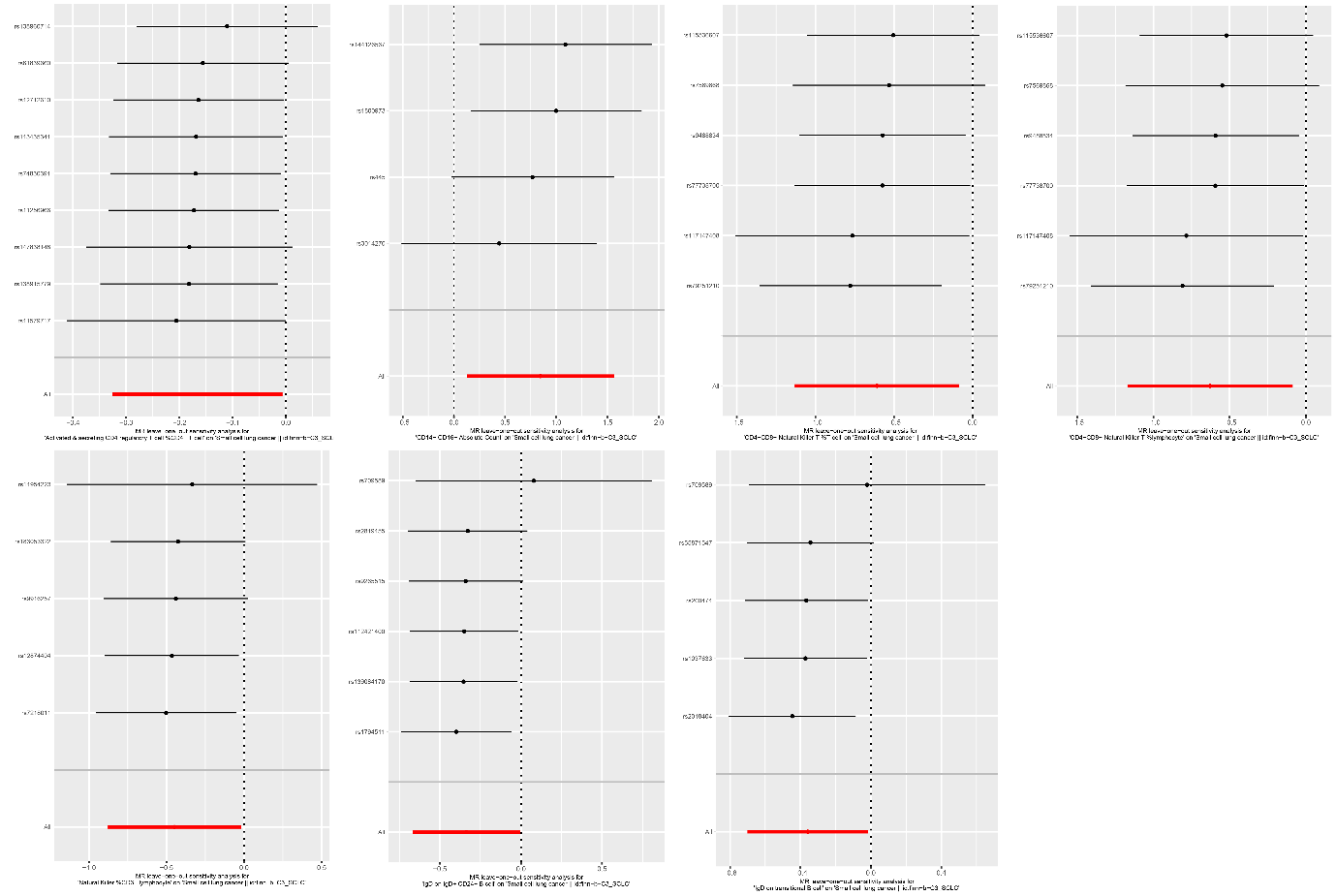

Supplement: Supplementary file 3 [file medi-104-e42053-s003.docx]
